# Help-seeking, trust and intimate partner violence: social connections amongst displaced and non-displaced Yezidi women and men in the Kurdistan region of northern Iraq

**DOI:** 10.1186/s13031-020-00305-w

**Published:** 2020-08-28

**Authors:** Alison Strang, Oonagh O’Brien, Maggie Sandilands, Rebecca Horn

**Affiliations:** Institute for Global Health and Development, Queen Margaret University, Edinburgh, UK

**Keywords:** Violence against women, Intimate partner violence, Gender-based violence, Conflict, Displacement, Social connections, Trust, Social capital, Faith, Religion

## Abstract

**Background:**

Conflict and displacement impact the social fabric of communities through the disruption of social connections and the erosion of trust. Effective humanitarian assistance requires understanding the social capital that shapes patterns of help-seeking in these circumstances - especially with stigmatised issues such as violence against women (VAW) and intimate partner violence (IPV).

**Methods:**

A novel social mapping methodology was adopted amongst a Yezidi population displaced by ISIS (ISIS: Islamic State of Iraq and Syria, locally known as Da’esh) occupation and a neighbouring settled Yezidi population in the Kurdistan region of northern Iraq in late 2016. Six participatory workshops were conducted to identify available resources with respect to: meeting basic needs, dispute resolution and VAW. Subsequently, 51 individual interviews were conducted (segmented by gender and settlement status) to identify connectedness to, and trust in, the resources identified, with a focus on IPV against women.

**Results:**

90% of participants reported God as a key source of help in the previous 6 months, representing the most widely cited resource. Following God, the most accessed and trusted resources were family and community, with NGO (non-governmental organisation) provision being the least. Women drew more strongly upon familial resources than men (*Χ*^2^ = 5.73, df = 1, *p* = 0.017). There was reduced trust in resources in relation to seeking help with IPV. A distinction between trust to provide emotional support and trust to resolve issues was identified. Settled women were 1.6 times more likely to trust community members and government services and 3.7 times more likely to trust NGOs than displaced women.

**Conclusions:**

Mapping social connections and trust provides valuable insight into the social capital available to support help-seeking in populations of humanitarian concern. For these Yezidi populations, family, religious and community resources were the most widely utilised and trusted. Trust was mostly reserved for family and their main religious leader regarding IPV against women. Lack of trust appeared to be a major barrier to stronger engagement with available NGO provision, particularly amongst displaced women. The role of faith and religious resources for this population is clearly significant, and warrants an explicitly faith-sensitive approach to humanitarian assistance.

## Introduction

From 2014, the population of Iraq faced significant violence, loss and displacement at the hands of groups associated with the Islamic State of Iraq and Syria (ISIS), locally known as Da’esh. The United Nations saw actions against the Yezidi population in particular as constituting genocide [1]. Alongside widespread killings, the Yezidi experienced the kidnapping of women, many of whom report being kept as sex slaves, the coercion of boys and men into the military, and forced religious conversion to Islam [2, 3].

Conflict and displacement impact the social fabric of communities through the disruption of social connections and the erosion of trust. These elements of social capital are crucial to the wellbeing of communities in terms of identity as well as social and economic activity. They are thus frequently the explicit focus of strategies to undermine community capacities. This was clearly the case with ISIS actions against the Yezidi communities of northern Iraq. However, a corollary of disruption of social capital being widely targeted as a measure to undermine community identity and functioning is an awareness that rebuilding social capital is a crucial element of community recovery. Understanding how to strengthen social capital, and how to work with the residual and evolving forms of such capital that shape patterns of help-seeking post-conflict, is a crucial task for humanitarian assistance.

Social capital, in its most basic terms, refers to the networks of relationships among people who live and work in a particular society, enabling that society to function effectively and includes the information, trust, and norms of reciprocity inhering in one’s social networks [4]. Social capital is built among individuals, and at community and societal levels through formal and informal institutions to create stable linkages, networks and trust [5]. Therefore, social connections or networks and trust (both personal and institutional trust) are integral elements of social capital, and characterised by reciprocity of exchange of resources [6, 7].

While there have been critiques of the concepts in the wider literature [5, 8–11], thinking in terms of social capital, social connections and trust have found particular resonance in work with conflict-affected and displaced communities. People in such circumstances are separated from their social connections, relationships of trust are undermined and thus social capital is depleted [12, 13]. Rebuilding social capital – including through re-establishing ‘bonds’ with co-ethnic or co-religious groups, forging ‘bridges’ with other communities, or enabling ‘links’ with civic and institutional structures – provides a framing agenda for processes of integration and (re)settlement [14]. However, most crucially in relation to the current paper, social connections provide a basis for populations to seek help and support to foster recovery.

Understanding the current coping and help-seeking strategies of affected communities is now widely considered a pre-requisite for effective humanitarian response [15–17]. A critical implication of the principle of localisation for humanitarian programming is the greater recognition of local capacity, initiatives and agendas, with interventions designed to bolster and support existing local strategies rather than displace them. The resources available, trusted and utilised by communities in their recovery efforts may differ widely depending on the nature and sensitivity of the challenges that are being addressed. Understanding these requires methodologies that are easy to implement and are not burdensome on vulnerable populations, nor the organisations seeking to support them.

Yezidi who were forced from their homes as a result of the military actions of ISIS, fled to the Duhok area in the Kurdistan region of northern Iraq. The Yezidi identify as a religious minority of the Kurdish peoples, and as such they sought the protection afforded by the government of the Kurdish autonomous region. Many settled either within or nearby existing Yezidi villages. Despite support from the local population and regional government, the displaced Yezidi population were struggling to meet basic needs such as shelter, security and food, and the disruption of livelihoods. Additionally, given the mounting evidence of enhanced risk of gender-based violence (GBV) amongst conflict-affected populations [18–22], there was significant concern regarding intimate partner violence (IPV) and related forms of abuse. Response to these needs by international humanitarian actors required some appraisal of the existing resources being brought to bear by the local populations on such challenges.

## Methods

This study reports deployment of a novel social mapping methodology in late 2016 amongst the Yezidi population living in adjacent, formal and informal settlements in the Duhok area of northern Iraq, proximal to the northern border of territory that came under ISIS control in 2014. A Yezidi village in the Duhok area was the focus of recruitment of the settled population. The informal settlement of tents and huts located at the outskirts of this village was the focus for recruitment from the displaced population, comprising Yezidi families who had fled ISIS controlled areas in the preceding 2 years. These locations were identified jointly with our field partners, Tearfund, from locations where they were already providing services. The selection enabled the study to compare the experiences of the settled (but war-affected) Yezidis with those of Yezidis recently displaced and living in informal settlements. At the same time, it ensured the research was conducted in a context where support would be immediately available to participants if needed and where research outcomes could be used to the benefit of the participating communities.

Six participatory workshops were conducted to identify resources available to this population (two with displaced women, two with displaced men, one with settled women and one with settled men). Separate workshops were held with the displaced and settled cohorts in order to observe the range of resources perceived as valued in each context, and to examine the different help-seeking pathways of both groups. Separate workshops were held for men and women to respect local cultural practice precluding the gathering of women and men together in public and to allow differentiated, gendered perspectives to emerge. It was not possible to conduct the intended set of eight workshops (2) for each cohort: settled women/settled men/displaced women/displaced men, due to limited availability of the settled community. However, previous experience^1^ in using the method suggested that this number of workshops would be sufficient to reach ‘saturation’ [23] in the generation of a core list locally of valued social resources. This was confirmed by the repeated emergence of the same social resources across the workshops. Had this proved not to be the case (with data sets looking very different from each workshop) further workshops would have been conducted until saturation point was reached.

The workshops were arranged in liaison with community leaders and the Tearfund Beneficiary and Accountability Officer. Participants were invited by the community leaders and through snowballing within the populations resulting in convenience sampling. Adults, volunteering to take part, were verified by the community leaders as either settled or displaced according to the agreed criteria, and allocated to the appropriate gendered workshop. The workshops – each engaging between eight and twelve adults - were held in spaces familiar to participants. In the informal displaced Yezidi settlement these were the living spaces of the local leader (*mukhtar*) and his family, which the family vacated for the duration of the workshops. In the Yezidi village the workshops were conducted in the local community centre.

Participatory workshops taking about 1 hour were facilitated by a trained local researcher, supported by one or two note-takers of the same gender as workshop participants, whose responsibility was to capture verbatim quotations from ongoing discussion. The workshops were observed by study authors (supported by discreet simultaneous translation). The purpose of the research was first outlined, and it was emphasised that participation was voluntary and that anyone was free to leave at any time. Discussions followed a structured protocol [24] adapted for this study in consultation with an advisory group comprising key stakeholders including service providers, local academics and community representatives. Three scenarios were used to consider resources available to meet basic needs such as food, medicine or baby supplies, to support a woman experiencing violence and to resolve disputes within and between communities. These were chosen and shaped by the advisory group to represent important challenges faced by the local Yezidi population. During the workshops participants were asked to identify relevant and available resources by addressing specific scenarios of the form: ‘If you or someone like you faced XXXX, who could they speak to about this? Who could they ask for help?’ On completion of all the workshops, a consolidated list of resources identified by two or more participants across the responses to all scenarios and including data across all the groups was compiled. This range of resources was used as a proxy for the full range of resources potentially available to the population. Individual cards representing each of these 35 resources were then produced.

Within 1 week of the workshops, confidential individual interviews were conducted with 13 displaced women, 14 displaced men, 11 settled women, and 13 settled men. A convenience sample, within the inclusion criteria of the study (over 18 years and living in either the village or informal settlement) was accessed through a snowballing process. Everyone who had taken part in the workshops was invited to take part in the interviews. In addition workshop participants were invited to put the research team in contact with other people they knew who fitted the criteria. Participants were again mobilised through the Beneficiary and Accountability Officer of Tearfund and the community leaders. The interview format was designed to require minimal time burden on participants and require minimal interview skill or training of the interviewer. Like the workshops, it followed a structured protocol [24] adapted for this study in consultation with the advisory group. The formulation of introductory text and questions was discussed, translated and back-translated to ensure that language was appropriate and sensitive, but that no meaning was lost. The focus of interviews was to assess connectedness to, and trust in the resources identified. The reference period for recall was the previous 6 months, defined as the period since the Yezidi ‘Red Wednesday’ New Year celebration. Participants completed three card sorts of the pile of resource cards.

First, participants were shown each card in turn, the name on the card was read out loud and the participant asked to indicate whether they had spoken to or asked this person or organisation for help in the last 6 months by putting the card on a pile for ‘Yes’ or ‘No’. Once this first sort was completed, participants were shown three line drawings of cups to provide a visual analogue scale to represent the extent to which they trusted a named person or organisation: ‘a lot’ (cup full); ‘a little’ (cup half full); and ‘not at all’ (cup empty). They were then shown each card in turn once more, with the name on the card again read aloud, and were invited to place the card below the relevant drawing. If a participant reported that they did not know enough about the item to make an appropriate judgement, the card was put to one side and coded as ‘not applicable’. The third and final card sort followed the same format as the second, but narrowed the question to address the extent to which they trusted the named resource in relation to providing assistance in the specific context of violence against a woman in the home. Women were asked the question: ‘If you were experiencing violence at home, how much would you trust this person or organisation to try and help you?’ Men were asked: ‘If a woman in your family was experiencing violence at home, how much would you trust this person or organisation to try and help her?’ The initial question about VAW which was asked in both the women and the men’s workshops, deliberately phrased the question in a very open manner. However, the data that emerged from the workshops demonstrated dominant concern about violence against women in the home by an intimate partner (IPV). After further consultation with the advisory group, it was therefore decided to focus the third interview question on trust in relation to IPV against women. In this way, comparative data on each of the connections identified by the workshops was collected. Interview participants were not asked to add further suggestions of potential resources as this would have added to the time burden required of participants, and be likely to duplicate the workshop data.

All responses were recorded on a reporting matrix for each participant, which included a section to record verbatim elaborations or discussions of participants’ card sort decisions. Given the categorical nature of data collected, subsequent statistical analysis was restricted to non-parametric methods. The connectedness score represents the number of people reporting connection with each of the resources during the previous 6 months. These have been combined to form five categories of resource type and the scores aggregated. Variation in frequency of reported connectedness to resources was analysed through computation of chi-square. Trust scores on specified resources were aggregated and expressed as a percentage of maximum trust score attainable, to facilitate cross-category comparison. Differences in individual trust scores by category – disaggregated by gender and settlement status – were analysed using MANOVA, with data transformed to z scores to ensure normal distribution. Thematic coding of verbatim quotes captured in the course of participatory workshops and individual interviews was the principal means of consolidating qualitative analysis. The analysis was guided by the principles of qualitative data analysis outlined by Creswell [24]. An inductive approach was taken to allow dominant themes to emerge from the data [23]. Themes were then reviewed by the research team, and the data re-categorised according to themes and sub-themes identified by the research team. Throughout the analysis emerging theory was discussed and refined with key stakeholders. The outcomes of the qualitative analysis will form the focus of another paper, but are used in this paper where they contribute to the understanding of the mobilisation of social networks in help-seeking.

The results of this study were shared for validation and discussion in workshops in Duhok and Erbil, with local stakeholders including community leaders, local NGOs, government, UN and humanitarian agencies.

## Results

### Awareness of resources

**Fig. 1:**
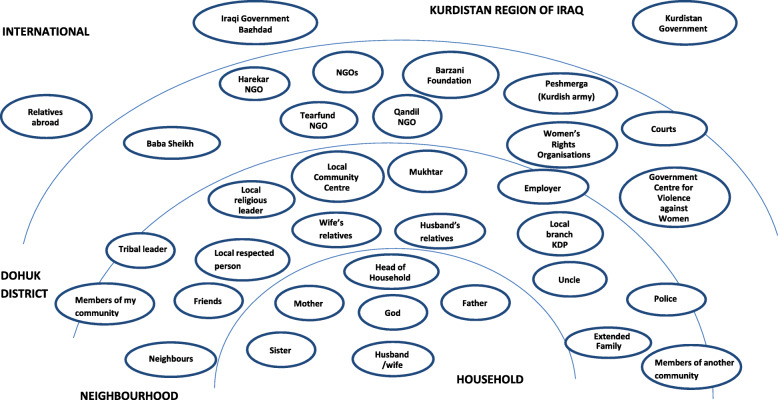
People and organisations identified by participants as available resources

Figure 1 shows the consolidated listing of resources identified through participatory group discussions, grouped by perceived proximity to the household.

Of these resources, the one that was most frequently called upon was God. 90% of participants reported that they had spoken to or asked God for help in the past 6 months. For further analysis, resources were grouped into one of five thematic categories (with the number of discrete resources coded to that category indicated in parentheses): family (ten), community (eight), government (eight), NGOs (five) and religious (three). Families were multi-generational, with extended family members commonly living together in the same neighbourhood before the conflict–driven displacements. A number of community structures were in place in the area, with each neighbourhood having a Mukhtar, a male leader appointed by local authorities to liaise between the community and external agents. Both displaced and settled Yezidis made use of a local ‘Cultural and Social Centre’ for community events and meetings. Participants principally represented the government of Kurdistan as a source of financial support either as employer or through grants for displaced people. Some participants reported receiving money from the Iraqi government in Baghdad because they had been employed by the state before they fled in 2014. Other political associations, such as the Barzani Foundation, the personal charitable fund of Kurdistan’s President, or the local party branch were identified as sources of help for meeting basic needs, as were the Peshmerga (Kurdish Army). Awareness of NGOs present in the area was limited, although groups did identify Tearfund, Harekar, Qandil, and a number of women’s rights associations as active. In terms of religious resources, the proximity of the Yezidi temple was reported as one of the key factors encouraging the displaced to have settled adjacent to the existing Yezidi settlement. Women reported gathering together there to pray if anxious about a specific issue. The Yezidi religious leader, Baba Sheikh, was cited as a resource to help resolve problems, being represented as both an approachable ‘comforter’ as well as the voice of authority in the context of disputes.

### Connectedness

**Fig. 2:**
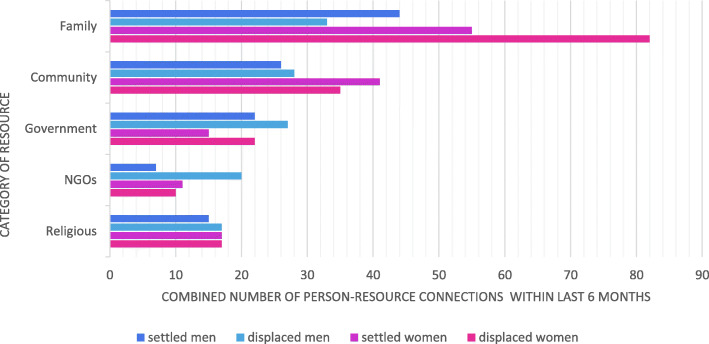
Aggregate number of people reporting connection with each resource by category

Figure 2 shows the aggregate number of people reporting connection with each of the resources within the five categories, disaggregated by gender and settlement status.

Familial and community resources were for all groups reported as the most accessed and NGO provision the least accessed. Women – and especially displaced women – drew more strongly upon familial resources than men (*Χ*^2^ = 5.73, df = 1, *p* = 0.017). Participants’ perspectives on help-seeking considered not just practical, material help or services, but also psychological support. Participants frequently referenced emotional support from family members:

*I depend on my parents so much for everything. I talk to my father about everything. He never lets me down*. [Displaced woman interview]

Financial support could be accessed from within the family or wider community. There were several reports of community collections to support individuals with a specific financial need, for example the collection held to pay a ransom for the daughter of a local Yezidi family held captive by ISIS. Another participant reported:

*We go to the tribal leader or richest person...there is one specific man who is very rich and he helps the people from the village or anyone who needs help*. [Settled women’s workshop]

However, economic conditions meant that it was sometimes impossible for family members to meet usual expectations of mutual aid:

*If I have money and you need money from me you can come to my home, and claim some money. I could sell something, I will sell my gold, my sheep, and I will collect money from people for you. Whatever I have for you I can give you to help you, but now I can’t do that.* [Displaced man interview]

Resources could be solicited from outside the community – for example through a Yezidi Facebook group - but members of the settled Yezidi population were more than three times more likely to be in contact with relatives abroad than those that were displaced.

### Trust

**Fig. 3:**
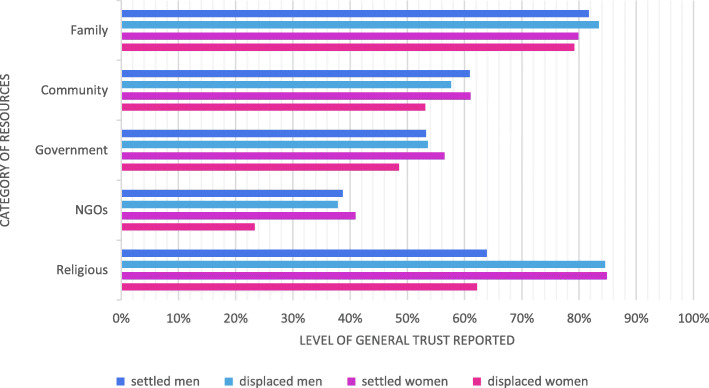
General levels of trust in each resource by category

Figure 3 presents participants’ reported levels of general trust in resources across the specified five categories, disaggregated by gender and settlement status.

**Fig. 4:**
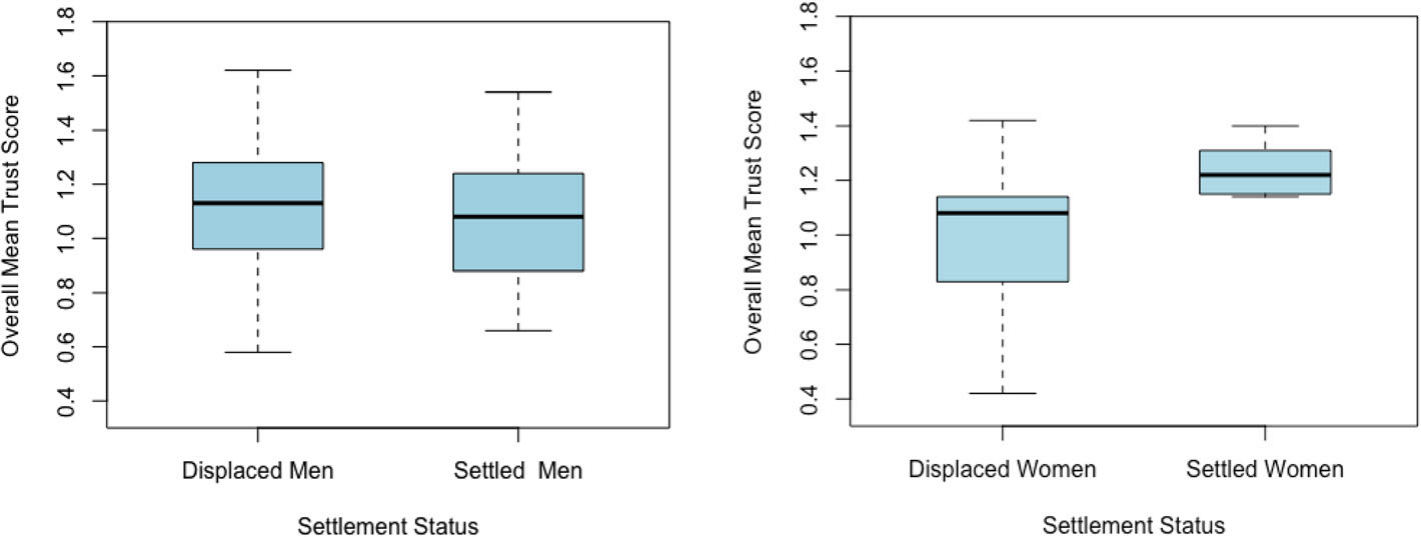
Distribution of average trust scores disaggregated by gender and settlement status

Overall, more of the Yezidi populations reported trust in familial and religious resources than in the provision by NGOs. Average levels of general trust reported by individuals across categories of resource were not significantly associated with gender or displacement status (F (5, 43) = 0.09, *p* > .05 and F (5, 43) = 0.089, *p* > .05 respectively). However, the overall distribution of scores suggested some interaction of gender and displacement status: displaced women were the least likely to report trust in resources across all categories and the inter-quartile range of their trust scores was non-overlapping with settled women (see Fig. 4).

Analysing at the level of discrete resources, men and women shared perceptions of which were the most trusted (i.e. God, immediate and extended family, and the Yezidi religious leader Baba Sheikh, all scoring above 75% of a maximum potential trust score). The authority of family and religious leaders was reinforced in participatory workshop discussions:

*If there are arguments, we try to bring the two families together to solve it. If not, we can go to the tribal leader or neighbours or anyone who is trustworthy…. All the people go to the older people and spiritual council, 99% of the problems are solved with the spiritual council. It has the highest place in our community and we cannot disobey their orders and whatever they say we have to obey them.* [Settled women’s workshop]

*One of the IDPs*^2^*was by the dam and was drunk, and was being rude and cursing people. Some men came and beat him to make him stop. He then sued them in court for 15 million Iraqi Dinar. They said they could not afford that so they went to Baba Sheik and he resolved the issue*. [Settled men’s workshop]

Otherwise, women showed greatest trust in their male relatives (81%), friends (79%), a specific local charitable foundation providing material support (74%) and the police (73%); men did so in the government (79%), their employer (78%), their uncles (77%), and the Peshmerga (76%). Displaced women reported lower levels of trust than all other groups.

**Fig. 5:**
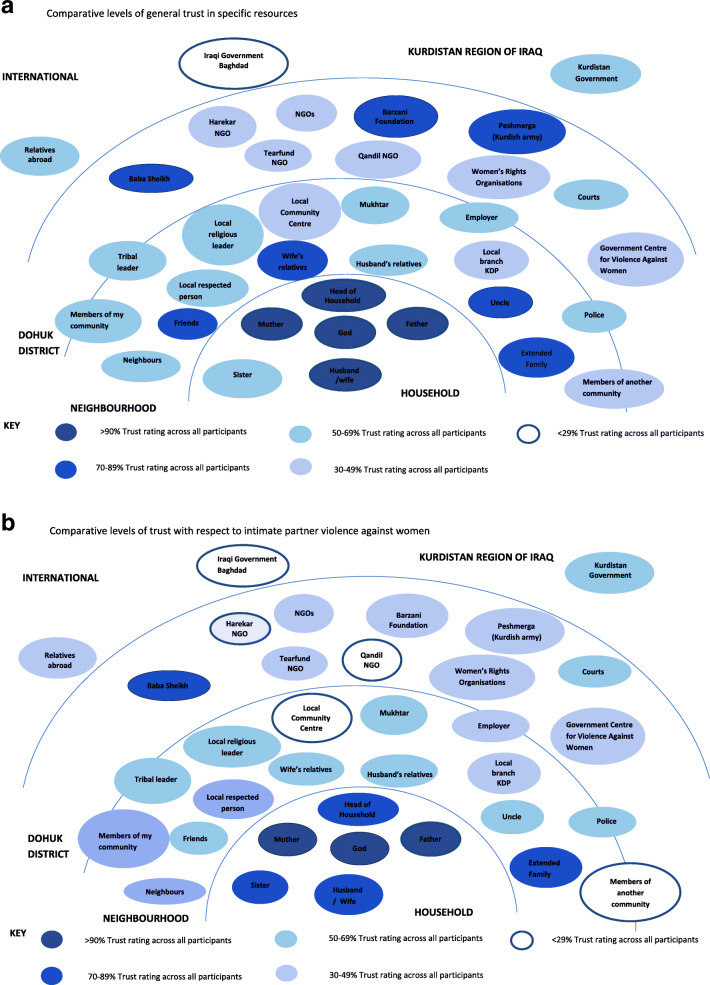
**a** Comparative levels of general trust in specific resources. **b** Comparative levels of trust in specific resources with respect to intimate partner violence against women

Figure 5a and b reflect reduced trust in resources in relation to the issue of IPV against women, compared to general levels of trust. For all groups there was a tendency to trust this issue within family networks and with religious resources, and far less with NGO, community or governmental resources.

In line with wider literature [25–27] our data demonstrated that Yezidi women in this study, commonly reported wishing to keep violence within the home a private domestic matter:

*It happened to me, my husband beat me, we solved it between us - we don’t want to make problems bigger than they are. Between us we solved it - we don’t want anyone to know our issues. In our tradition it is better not to share these things with strange people.* [Displaced woman interview]

There was a clear sense from participants that speaking about violence within a marriage is likely to make things worse rather than better. Kennedy [28] identifies perceptions of the benefits and quality of help as a key stage in help seeking amongst female survivors of sexual abuse. The identity of being an IDP appeared to make some women feel even more vulnerable about wider disclosure:

*The violence is happening but I tell myself it is not worth it and I try to keep silent. He beats me but we are IDPs, it would be better to keep silent than making the problem bigger than its normal size.* [Displaced woman interview]

The precariousness of the lives of these displaced Yezidi women would be likely to mean that stigma, potentially leading to divorce, and exclusion from future marriage is perceived as an impossibly high price to pay – even for the sake of personal safety [27, 29].

For the majority of women and men in this study, reporting violence in the home was seen as appropriate in certain circumstances, but the channels of doing so were clearly prescribed. Our data points to two distinct types of help valued by the women: emotional support, and practical help. For women, mothers were often the first point of recourse:

*When I cannot stand my miserable life then I go to speak to my mother to feel comfortable. I feel safe and free when I speak with her*. [Displaced woman interview]

*My mother… in any situation possible I ask her for help. She is my friend. My mother helps me to feel confident about myself.* [Settled woman interview]

Women reported speaking with their mothers and also other women as a source of emotional support, rather than resolution of the violence. Women also valued the comfort of private prayer and prayer with other women.

Male relatives were more likely to seek a resolution through wider consultation in the family:

*If I had a problem with my wife my father would be the best person to talk to. He’s the person we would listen to whatever he says he is the image of the family* [Displaced man interview]

*The father would talk to the husband’s relatives and ask them to talk to their son. The parents would talk to their son and that would resolve things.* [Settled woman interview]

When family discussions could not resolve ongoing violence in the home, the most favoured recourse would be to religious authority. For example, one mother addressing violence against her daughter noted:

*First I go to my daughter and try and resolve it with my husband. Then we go to the head of the household. If that doesn’t work, we go to Baba Sheikh…* [Displaced woman interview]

Another woman noted:

*If it is just verbal and daily arguments it is fine, but if it is serious and my family can’t solve it then we go to the religious people… If my husband wanted to divorce because he wanted another wife, I would go to the Sheikh and ask for help. He would collect the man and talk to relatives and parents. If this doesn’t work, the Sheikh would lead them to court.* [Settled woman interview]

Whilst extended family members and religious leaders were seen as a resource for resolving disputes arising from violence within a marriage, there was wide reluctance to share reports of violence with government authorities such as the police, or NGOs. A common theme in justifying this reluctance was the sense of dishonour or shame that such reporting would bring upon the family. People were conscious of the potentially very serious economic and social consequences of bringing shame on the whole family:

*If a woman went there [named NGO] she would lose her reputation in the wider family. If she is married she might get divorced – her husband will divorce her. If it is a woman, unmarried, she will not get married.* [Settled woman interview].

*I wouldn’t go to the police at all, I disagree with that. I am shy and don’t want to get a bad reputation. I love my family. I don’t want them to hate me.* [Displaced woman interview]

Settled and displaced Yezidi women trusted family and religious resources far more than community, government or NGO resources with issues of domestic violence. Nevertheless, the trust scores demonstrate that settled women were 1.6 times more likely to trust community members and government services and 3.7 times more likely to trust NGOs on this issue than displaced women. The qualitative data suggests that trust in the context of IPV against women should be seen in at least two different ways: trust to provide emotional support, or trust to have the capacity to improve the situation. These two capacities may sometimes overlap (most likely in the person of the woman’s mother), but not always.

## Discussion

The findings of this study documented both important commonalities and pertinent differences between men and women, and between displaced and settled Yezidi. In terms of gender, the analysis highlighted – notwithstanding shared connection and trust in God and the family – key contrasts in the social worlds of Yezidi men and women. Women and men shared their perspectives of the sacred and the familial. However, in contrast with men, women generally extended their trust to friends and female extended family members, and saw the police and a local group working on women’s issues in the area as the resources beyond this circle that they could call upon. For men, male relatives such as uncles provided familial support. Beyond family members, it was employers and the government – including groups such as the Peshmerga – that could be trusted. These networks of trust signal important gender-sensitive avenues in addressing the needs and concerns of Yezidi women and men.

Using the example of help-seeking in response to IPV against women has opened up our understanding of the functioning of trust within a highly stigmatised issue. Whilst this study was not designed to investigate stigma, the data shows a dramatic reduction in the resources that are trusted to address IPV in this context. Analysis of the qualitative data suggests that women in particular, were weighing up a complex set of factors in deciding whether, and how to seek emotional support and potentially practical help in addressing their situation. It has been established in the literature that ‘victim-blaming’ is common and functions as a powerful barrier to help seeking for intimate partner violence [25, 30]. It may be that the preference to seek emotional support from a mother, or other close female relative or friend demonstrated in this study, reflects a ‘trust’, or expectation that disclosure would be met with understanding/sympathy rather than negative judgement. On the other hand, whilst some participants spoke about the potential mediating role of a mother, there was consistent reporting that resolving disputes normally depended on the intervention of either male relatives – or if this failed, the intervention of an authority figure within the community. This points to a different kind of trust, trust in the capacity of the person or organisation to improve rather than worsen the situation. As McCleary-Sills [25] points out, there are three core aspects of help that women survivors of IPV would benefit from: emotional support, help to secure physical safety and recovery, and justice. The multiple barriers preventing access to each of these is apparent in this study.

Differences in the social resources available to settled and displaced Yezidi populations highlighted the social disruption and depletion of social capital associated with recent occupation, conflict-exposure and displacement. Although the settled Yezidi community had been on the frontline of the conflict with ISIS, it was clear that this population enjoyed significantly greater access to key resources such as employment. In the social sphere, the clearest differences in reports from displaced and settled communities were with respect to women’s trust in institutions beyond the family. This was most marked with respect to seeking assistance in the context of IPV against women. The fact that settled women were much more likely than displaced women to trust community members and government services as well as NGOs on this issue indicates that assistance from these sources in the course of resettlement had not served to (re)build trust in these institutions beyond the family. A common recommendation in the area of violence against women and girls in conflict and humanitarian settings is to allocate sufficient resources to provide services that respond to these issues in a meaningful way [31]. Whilst this is important, it does not take into account the extent to which women trust and are willing to access these services. The current study emphasises the need to start from where women (and men) are in terms of their help-seeking behaviour. Further research to elaborate trust in relation to different types of help would provide a deeper understanding of the social resources that conflict-affected women and men are able to mobilise to address IPV against women. The provision of health, psychosocial and legal support services will be ineffective if potential users do not have trust in the providers’ capacity to deliver the support perceived as needed in a way that leads to an overall reduction of harm.

A striking and recurring commonality signalled throughout the data collection was the importance of religious practice and of God as a source of help. Women spoke about the comfort of private and collective prayer. It may be that the privacy of their religious practice gave emotional support by providing an avenue of expression of distress whilst ensuring the avoidance of blame. There is increasing recognition of the role of religious coping and other forms of support seeking among conflict-affected civilians in low and middle income countries [16, 32]. Until recently there has been little attention paid to mechanisms of humanitarian engagement that respect and utilise these resources where appropriate. The recent articulation of principles for a ‘faith-sensitive’ approach to humanitarian assistance [33] – recognising and building upon religious resources valued and exercised by affected populations – is clearly pertinent for developing humanitarian strategy in contexts such as that focused upon here. A recent summary of the current evidence-base in relation to violence against women and girls in conflict and humanitarian settings identifies community-based approaches utilising faith leaders as a promising practice [31]. However, a study of community-based approaches that targeted faith leaders in the Democratic Republic of the Congo as service providers [34] noted that,

“Whilst most respondents believed that their religious institution supported survivors (74%), only 11% of survivors felt that a faith leader was able to provide effective support.” (p22)

Whilst the current study suggests that faith leaders are in a good position to support survivors of gender-based and other forms of violence, due to the high level of trust women have in them, more research is needed into the support that faith leaders are able to provide to survivors in these contexts, and crucially, how to avoid doing harm. Furthermore, given the emphasis placed on private prayer over formal religious structures by women in this study, the value of personal religious belief and practice should not be ignored.

### Limitations

The study focused on two specific Yezidi communities in the proximity of Duhok and caution needs to be exercised in generalising findings from these settlements to other Yezidi communities in the Nineveh governorate and beyond. Sampling within communities was opportunistic and so did not access a representative sample of the targeted populations. As a mixed methods design with a modest sample size, the study was not powered for parametric, multivariate analysis.

## Conclusions

Undertaking sensitive research in a precarious conflict zone with much movement of population presents multiple practical and ethical challenges. As a result, there is a dearth of understanding about the coping and resilience of conflict-affected populations. The findings in this study indicate that a participatory social connections mapping process can usefully identify patterns of social connection and trust that influence help-seeking behaviour in a humanitarian setting, and thus inform the design of more effective and appropriate humanitarian intervention strategies.

This approach could also have wider application within the humanitarian sector, particularly in supporting accountability and the localisation agenda, since it enables an increased understanding of the affected community’s perspectives, priorities, capacities and resources, rather than simply focusing on their needs. This process could be used to strengthen humanitarian response service mapping, to ensure triangulation with community perspectives and to help identify resources and address barriers. It can also help reveal different perspectives within populations, encouraging gender-sensitive programming, and consideration of the impacts of displacement.

This study emphasised how trust remains a vital factor in terms of access to services, even where these are available, and particularly for sensitive issues such as IPV against women. It has highlighted the value and importance of emotional support as well as practical service provision, and suggested that more attention needs to be paid not only to levels of trust, but also types of trust in potential social resources. The mapping demonstrated a low level of awareness of NGO provision amongst the local community, accompanied by low levels of trust, but highlighted the importance of local faith resources to the affected population. This indicates the need for a more faith-sensitive approach to humanitarian assistance. Existing trusted networks must be effectively mobilised in order to provide meaningful and accessible support in the short term. Those same networks, in turn, will be the most effective means to shape attitudes and address stigma in the long term.

## Data Availability

The datasets used and/or analysed during the current study are available from the corresponding author on reasonable request.

## Abbreviations

GBV: Gender-based violence
IPV: Intimate partner violence
VAW: Violence against women
IDP: Internally displaced person
ISIS: Islamic State of Iraq and Syria
NGO: Non-governmental organisation
UN: United Nations

## Footnotes

1 The method was originally piloted by the QMU research team amongst internally displaced persons in Darfur in 2013.

2 IDP: Internally displaced person
